# Epigenetics Underlying Susceptibility and Resilience Relating to Daily Life Stress, Work Stress, and Socioeconomic Status

**DOI:** 10.3389/fpsyt.2020.00163

**Published:** 2020-03-20

**Authors:** Michael G. Gottschalk, Katharina Domschke, Miriam A. Schiele

**Affiliations:** ^1^Department of Psychiatry and Psychotherapy, Medical Center – University of Freiburg, Faculty of Medicine, University of Freiburg, Freiburg, Germany; ^2^Center for Basics in NeuroModulation, Faculty of Medicine, University of Freiburg, Freiburg, Germany

**Keywords:** epigenetics (MeSH), work stress, daily life stress, socioeconomic status (MeSH), susceptibility, resilience (psychological), candidate risk variants, epigenome wide association

## Abstract

Susceptibility and resilience to mental disorders result from a complex choreography of gene–environment interactions with epigenetics at the intersection of external psychological stressors and internal biological systems. Increasing awareness of the growing disease burden influenced by daily life stress (“daily hassles”), work-related stress, and low socioeconomic status (SES) has resulted in a novel interest into their underlying molecular signatures. This review offers a brief outline of psychiatric epigenetics and a comprehensive overview of recent findings exploring the relationship of various occupational stressors and DNA methylation in epigenome-wide association studies (EWAS) and in candidate gene studies including the serotonin transporter (*SLC6A4*; *5-HTT*LPR), melatonin receptor 1A (*MTNR1A*), brain-derived neurotrophic factor (*BDNF*), tyrosine hydroxylase (*TH*), and the protein family of DNA methyltransferases (*DNMTs*). Conceptual and methodological challenges of epigenetic investigations with a special focus on gene–environment interactions are highlighted and discussed. The findings are integrated into a pathophysiological framework featuring epigenetic plasticity factors and work-related stress as a possible central detrimental component targetable by workplace interventions. Finally, the potential of dynamic epigenetic biomarkers of treatment response to pharmacotherapy or psychotherapy is expanded upon.

## Introduction

Chronic exposure to environmental stressors, including but not limited to work-related stress, has been implicated in the patho-etiology of various mental disorders and represents a substantial cause of morbidity, imposing a considerable societal disease burden (1). An individual’s response to detrimental environmental influences is affected by an interplay of its intrinsic genetic configuration and its susceptibility or resilience toward external stimuli, which has led to a growing understanding of gene–environment interactions in the onset, course, and treatment of mental disorders (2). Epigenetics has emerged as a molecular correlate of this intersection, and a converging body of evidence has been collected outlining the effects of early-life stress and traumatic experiences, but less so of chronic psychosocial stressors on the expression of candidate genes involved in psychopathology (3).

Given the known connections between increased work-related stress and poor long-term mental health outcome, this mini review explicitly focuses on summarizing the current state of the literature on the epigenetics of daily life stress, work-related stress, and socioeconomic status (SES) and highlights future challenges and opportunities relevant to the field of psychiatry and psychology. In order to offer a concise overview, PubMed searches combining the terms “work/daily/occupational stress” and “socioeconomic status” with “epigenetics” and “DNA/genome wide methylation” were performed in October 2019, and the resulting titles and abstracts were screened for relevance. Please refer to **Table 1** for an overview of primary findings.

**Table 1:** Overview of epigenetic studies relating to daily life stress, work stress, and socioeconomic status.

Study	Sample	Tissue	Assessment	Gene(s)/scope	Findings
***Daily life stress***					
**Duman and Canli** (4)	105 Caucasian males	Blood	TICS for past 3 months	*SLC6A4*	↑ total methylation as a function of chronic daily stress in *5-HTTLPR* l/l genotype carriers ↑ promoter methylation as a function of chronic daily stress in *5-HTTLPR* s allele carriers
**Pishva et al**. (5)	112 Dutch/Belgian psychiatrically healthy individuals, a population-based replication sample of 434 individuals, 85 unaffected siblings of patients with psychosis, 110 patients with psychosis, 126 patients with a MDD lifetime history and residual symptoms	Blood, saliva, or buccal epithelium	Experience sampling method for daily stressful and pleasant events	*DNMT1*, *DNMT3A*, *DNMT3B*, *MTHFR*	Association of the minor *DNMT3A* rs11683424 T allele with a reduced impact of daily life stress on negative affectivity in the discovery sample, replication sample, and in the sample of patients with a MDD lifetime history
***Work stress***					
**Alasaari et al**. (6)	24 Finnish female nurses reporting high and 25 female nurses reporting low work stress environments	Blood	JCQ	*SLC6A4*	↓ promoter methylation associated with increased perceived work stress
**Miyaki et al**. (7)	360 Japanese manufacturing company workers	Saliva	JCQ	*TH*	↑ overall average / promoter / 5’-regulatory region methylation associated with increased work strain
**Song et al**. (8)	360 Japanese manufacturing company workers	Saliva	JCQ	*BDNF*	↑ overall average methylation associated with increased work strain
**Sulkava et al**. (9)	59 Finnish pilots, flight attendants, and nurses (from Alasaari et al., 2014)	Blood	–	*MTNR1A*	↑ methylation levels in the *MTNR1A* promoter region associated with the minor *MTNR1A* rs12506228 A allele (discovered in a GWAS of work-related exhaustion in the same study)
***SES***					
**Bush et al**. (10)	178 kindergarten children from mixed ethnic backgrounds	Buccal epithelium	Composite measure of parental income and education used as a proxy of SES	EWAS	Overrepresentation of altered methylation at CpG sites in genes related to immune and developmental regulation functions associated with low SES
**Coker et al**. (11)	241 Mexican-American maternal–infant pairs	Cord blood	Neighborhood-level characteristics used as a proxy of SES	EWAS	↑ methylation of LINE1 elements associated with low SES
**Fiorito et al**. (12)	Meta-analysis of 5,087 samples of independent cohorts from Australia, Ireland, and Italy	Blood	Highest level of educational attainment used as a proxy of SES	Accelerated intrinsic epigenetic aging	Increased accelerated epigenetic aging associated with low SES
**Jones-Mason et al**. (13)	100 late adolescents from mixed ethnic backgrounds	Blood	Hollingshead Measure of SES	*SLC6A4*	↑ methylation and low SES linked to increased reports of trauma
**Laubach et al**. (14)	609 maternal–infant pairs from mixed ethnic backgrounds	Cord blood and peripheral blood (3-year follow-up)	Composite measure of individual- and neighborhood-level metrics used as a proxy of SES	EWAS	Altered methylation in CpG sites mapped to *ACSF3*, *TNRC6C*-*AS1*, *MTMR4* and *LRRN4* associated with low SES, *LRRN4* methylation changes persisted into early childhood
**McDade et al**. (15)	489 Filipino youth	Blood	Composite measure of income, assets, and education used as a proxy of SES	EWAS	Overrepresentation of altered methylation at CpG sites in genes related to immune functions, skeletal development, and CNS development associated with low SES
**Santos et al**. (16)	426 infants born <28 weeks of gestation from mixed ethnic backgrounds	Placenta	Composite measure of parental education, marital status, food and nutritional service assistance, and public health insurance used as a proxy of SES	EWAS	Overrepresentation of altered methylation at CpG sites in genes related to cellular immune and stress response associated with low SES
**Swartz et al**. (17)	132 Caucasian adolescents	Saliva	Highest level of parental educational attainment used as a proxy of SES	*SLC6A4*	↑ methylation change over 2 years associated with low SES and increased threat-related amygdala reactivity
**Uddin et al**. (18)	77 trauma-exposed controls and 23 PTSD patients, primarily with African-American background	Blood	Highest level of educational attainment used as a proxy of SES	EWAS	Methylation×SES interaction occurred primarily in genes related to nervous system function

5-HTTLPR, serotonin transporter-linked polymorphic region; ACSF3, acyl-CoA synthetase family member 3 gene; BDNF, brain-derived neurotrophic factor gene; DNMT1, DNA methyltransferase 1 gene; DNMT3A, DNA methyltransferase 3 alpha gene; DNMT3B, DNA methyltransferase 3 beta gene; EWAS, epigenome-wide association study; GWAS, genome-wide association study; JCQ, Karasek’s Job Content Questionnaire; LINE1, L1 long interspersed nuclear elements; LRRN4, leucine-rich repeat neuronal 4 gene; MDD, major depressive disorder; MTHFR, methylenetetrahydrofolate reductase gene; MTMR4, myotubularin-related protein 4 gene; MTNR1A, melatonin receptor 1A gene; SES, socioeconomic status; SLC6A4, serotonin transporter gene; TH, tyrosine hydroxylase gene; TICS, Trier Inventory of Chronic Stress; TNRC6C-AS1, TNRC6C antisense RNA 1 gene. Arrows indicate increased (↑) and decreased (↓) methylation, respectively.

## A Brief Background in Epigenetics

Epigenetics constitutes the effects of DNA and histone modifications and of micro RNAs, subsumed as changes to the structure of the DNA strand (but importantly not as changes to the DNA sequence itself), or the interaction of mRNA with noncoding RNAs, affecting transcription and translation. Epigenetic modifications describe dynamic processes with an inherent capacity of intraindividual reversibility. The functionally most well described and major epigenetic modification with relevance to available studies investigating work stress-related phenomena is DNA methylation. Therefore, other epigenetic processes will not be further expanded upon. DNA methylation occurs at cytosine carbon atoms in CpG nucleotide pairs, often accumulating in CpG-rich genomic sites referred to as CpG islands (19). DNA methylations are catalyzed by the enzyme class of DNA-methyltransferases (DNMTs) and become functionally relevant due to other proteins recognizing and binding to methylated DNA sequences and thereby, for example, prohibiting other interaction partners of the transcription start complex to bind or pass, resulting in a *de facto* silencing of the downstream genomic region. Generally speaking, increased CpG methylation of promoters, enhancer elements, or transcription start sites leads to lower transcription levels of the associated mRNA, whereas decreased methylation of the respective sites would be linked to higher transcript concentrations (20). It should be noted that structural [e.g., single nucleotide polymorphisms (SNPs)] and functional (e.g., CpG methylation) changes of the DNA share an interdependence, insofar as for example, SNPs can interfere with target recognition sites of enzymes and hence affect the local occurrence of epigenetic base pair modifications, while methylation changes can alter the topological susceptibility to regional mutations, both of which may impact higher levels of biological complexity including cognitive processes and behavior (21, 22).

## Daily Life Stress and Epigenetics

The serotonin transporter-linked polymorphic region (*5-HTT*LPR), a promoter region polymorphism of the serotonin transporter gene (*SLC6A4*)—differentiated into a high transcription (more active) long (l) allele and a less active short (s) allele—as well as *SLC6A4* methylation have been commonly investigated in interaction with early life stressors on stress responsivity. Far fewer studies, however, have integrated the role of current stressful events. One such study not only evaluated the interaction between early life stress and *5-HTT*LPR genotype on blood-based *SLC6A4* methylation, *SLC6A4* expression, and cortisol response to the Trier Social Stress Test (TSST) but also analyzed the association of chronic stress experiences, gene expression, and cortisol response as a function of *5-HTT*LPR genotypes (4). Current stress experience over the past 3 months as measured by the Trier Inventory of Chronic Stress (TICS) and baseline *SLC6A4* expression in 105 psychiatrically healthy Caucasian males did not differ as a function of *5-HTT*LPR genotype, whereas l/l genotype carriers displayed significantly higher *SLC6A4* expression in response to the TSST, as compared to s allele carriers. Interestingly, l/l genotype carriers further showed increased global promoter methylation levels, both as a function of early-life stress [childhood trauma questionnaire (CTQ)] and in interaction with recent chronic daily stress. Upon closer investigation, s allele carriers, but not l/l genotype carriers, possessed significantly increased methylation of CpG islands flanked upstream by the 5-*HTT*LPR and downstream by the untranslated first *SLC6A4* exon, which showed increased methylation levels as a function of recent chronic daily stress and correlated positively with glucocorticoid receptor (*NR3C1*) expression [for more evidence of *NR3C1* as a key mediator of early adversity in stress-related disorders, please refer to Palma-Gudiel, Cordova-Palomera (23)]. Given that elevated *SLC6A4* methylation levels of the very same site have been linked to major depressive disorder (MDD) (24) and increased levels of burnout symptoms (6) (see below), this distinct genomic region might represent a particularly dynamic methylation site, responsive to chronic recent daily stressors.

By way of an indirect epigenetic approach, a multistep gene–environment interaction study tested for main and interaction effects of 31 SNPs in epigenetic regulatory genes and daily life stress or pleasant experiences, respectively, on emotional affectivity in a discovery sample of 112 psychiatrically healthy individuals, a population-based replication sample of 434 individuals and three further samples of unaffected siblings of patients with psychosis (n = 85), patients with psychosis (n = 110), and patients with a MDD lifetime history and residual symptoms (n = 126) (5). All samples showed associations between daily life stress, negative affectivity, pleasant experiences, and positive affectivity, respectively, yet no SNP main effect emerged for either negative or positive affectivity. Following *post hoc* multiple comparisons correction in the interaction analyses of SNPs and daily life stress or pleasant experiences, respectively, in the discovery sample, only one SNP was validated as significant and directionally similar in the replication sample and furthermore in the sample of patients with a MDD lifetime history: The minor rs11683424 T allele in the gene coding for the DNA methyltransferase 3 alpha (*DNMT3A*) was associated with a reduced impact of daily life stress on negative affectivity. This result is particularly relevant since DNMT3A is classified as a *de novo* methyltransferase, dynamically reshaping methylation patterns, rather than statically preserving preexisting methylation patterns during DNA replication, as for example maintained by DNMT1 (25), signifying that genetic variation in regulatory enzymes of DNA methylation might moderate the *ad hoc* effects of daily life stressors on emotional regulation and thereby psychopathological susceptibility and resilience *via* downstream epigenetic processes.

## Work-Related Stress and Epigenetics

Considering the adverse emotional and physical responses to chronic work-related stress, which are reached when the work demands exceed an individual’s coping ability and capacity (26), it appears surprising how seldom the molecular interplay of perceived occupational stressors and maladaptive outcomes has been examined.

In a female nurses cohort who reported either high (n = 24) or low (n = 25) work stress environments, significantly lower *SLC6A4* methylation of the same region described by Duman and Canli (4) above was discerned in the high-stress group (6). Notably, in a multifactorial analysis of covariance including work stress environment, burnout, work control, work demand, age, and *5-HTT*LPR genotypes as explanatory variables, only perceived work stress significantly and independently affected overall methylation levels. Higher methylation levels were associated with an increasing intensity of burnout symptom reports when corrected for perceived work stress and vice versa. It could be hypothesized that *SLC6A4* promoter hypomethylation reflects an adaptational mechanism independent of the *5-HTT*LPR genotype in response to work-related stress, which may initially follow a physiological role to minimize stress exposure and preserve cognitive functioning, but results in dysfunctional long-term consequences manifesting as failed coping mechanisms and increasingly depressed mood.

Another impactful factor of work-related stress is shift work, which has been linked to adverse long-term health effects, including, but not limited to, an increased risk of mental disorders (27), but whose molecular underpinnings have rarely been explored. In a fixed-effects meta-analysis of genome-wide association studies (GWAS) (total n = 753 samples, including subsets of the general population, nurses, and airline employees) of work-related exhaustion, rs12506228 was the only SNP reaching genome-wide significance (*p* = 4.90 × 10^-8^) (9). Since rs12506228 is located in a CpG island in the 5’-regulatory region of the gene coding for the melatonin receptor 1A (*MTNR1A*), a subsequent *in silico* search of available postmortem brain data suggested reduced *MTNR1A* central nervous system (CNS) expression levels to be linked to the minor »risk« A allele. Complementing this transcriptomic approach, an analysis of bead chip methylation data of pooled blood samples from the nurse (6) and airline employee sample subsets (total n = 59) further hinted at significantly higher methylation levels in the *MTNR1A* promoter region to be associated with the rs12506228 *MTNR1A* A allele. Reduced central melatonin signaling in rs12506228 A allele carriers, especially considering its inhibitory effect *via* MTNR1A in the suprachiasmatic nucleus, where melatonin acts as a stabilizer of the circadian rhythm against light-induced phase shifts (28), might result in an increased sensitivity to nocturnal lights, for example, because of epigenetically reduced expression of *MTNR1A*, leading to circadian disruptions and ultimately increased work-related stress due to night shifts.

Additional candidate gene approaches targeting the epigenetics of work-related stress have been conducted in a cohort of 360 Japanese manufacturing company workers, who were subdivided into four quartile-based groups according to their reported job strain. The overall average methylation across CpG sites covering the tyrosine hydroxylase gene (*TH*) was significantly elevated in workers reporting a higher work strain, as was the methylation of the promoter and the 5’-regulatory region by itself, with methylation and the degree of work-related stress levels displaying a significant positive correlation (7). Furthermore, the overall average methylation of the gene coding for the brain-derived neurotrophic factor (*BDNF*) was increased in individuals with higher reported work strain (8). These results highlight the need to account for the temporal dynamic of epigenetic modifications associated with work-related stress, for example, *BDNF* methylation has been demonstrated to be unaltered following acute psychosocial stress (29), while preclinical evidence proposes that chronic stress exposure leads to a glucocorticoid-dependent hypermethylation of *TH*, but only when combined with additional neuropsychiatric risk mutations (30).

## Socioeconomic Status and Epigenetics

Recently, interest has sparked into investigations of epigenetics related to SES, as a combination of low SES and high job demands has been linked to increased morning cortisol and diurnal cortisol secretion (31), supporting the notion that lower SES is associated with various work-related stressors precipitating a higher prevalence of mental disorders and health-impairing behaviors (32).

A meta-analysis of three samples (total n = 5,087) reported a linear increase of accelerated intrinsic epigenetic aging when comparing high to medium and low SES (12). Moreover, individuals who experienced a change in SES over their life span (based on the trajectory of father’s occupational position as a proxy of early-life SES and individual highest occupational position as a proxy for adulthood SES) showed intermediate levels of accelerated epigenetic aging, indicating a potential reversibility of maladaptive epigenetic effects due to low SES. Notably, sex-dependent DNA methylation changes associated with SES have been demonstrated to already exist at the time of birth and to partially persist into early childhood (14, 16), mainly affecting biological processes regulating immunological and developmental functions (10).

Integrating attachment variables into an analysis of SES and *SLC6A4* promoter methylation in 100 adolescents, higher methylation and low SES were linked to higher reports of trauma (13). Within the group with unresolved attachment style, yet not in the groups with secure or organized attachment style, low SES was associated with increased methylation, implying that attachment security might act as a protective factor against the effects of low SES on *SLC6A4* promoter methylation. This notion appears to be particularly relevant since an explorative epigenome-wide association study (EWAS) including trauma-exposed controls (n = 77) and post-traumatic stress disorder (PTSD) patients (n = 23) has proposed that methylation × SES interaction effects occur primarily in genes related to nervous system function (18), offering a biological pathway by which external stressors might elicit maladaptive central changes. Additionally, biological pathways highlighted by EWAS of early-life SES-related stress have pointed toward altered methylation in genes governing CNS and immune functions (10, 15), some of which appear stable over different developmental stages (14), and with a particular molecular focus on L1 long interspersed nuclear elements (LINE1 elements) hypermethylation (11).

Reasonably, continuous research efforts have been invested into an elucidation of how low SES results in a heightened risk of mental disorders. A longitudinal multilevel approach integrating epigenetic, neuroimaging, and psychometric readouts from 132 adolescents reported that low SES was the only factor among environmental stress variables predicting a greater 2-year increase in *SLC6A4* promoter methylation, independent of a MDD family history (17). Besides, increases in proximal *SLC6A4* promoter methylation predicted increases in threat-related amygdala reactivity over the same time window, which in turn predicted elevated levels of depressive symptoms another year later, but this time solely in individuals with a MDD family history. A moderated mediation model confirmed that lower SES indirectly affected future increases in depressive symptom intensity in individuals with, but not without, a MDD family history, *via SLC6A4* promoter hypermethylation, which in turn predicted heightened amygdala responsivity. These findings add to the notion of elevated peripheral *SLC6A4* promoter methylation as an early surrogate stress marker reflecting increased central amygdala reactivity and lower brain expression levels of *SLC6A4* (33).

## Discussion

### Conceptual and Methodological Challenges

As exemplified by the above studies, investigations into stress-related epigenetics not only have to reach adequate sample numbers to allow for the detection of potentially small effect sizes, they also have to specifically monitor the homogeneity of their cohorts, for example, sex (34), ethnicity (35), and smoking status (36) have all been shown to significantly influence the epigenome. Also, it should be kept in mind that, as with most human psychiatric research, the above listed findings constitute correlational evidence in need of further independent replication. Moreover, a notorious challenge is the division into epigenetic responses to recent environmental stressors and methylation changes that date back to conception. The epigenetic modifications resulting from *in utero* exposure or during critical “windows of vulnerability” in early-life development have been demonstrated to permanently affect methylation patterns for several decades (37, 38). Therefore, longitudinal within-subject designs should be preferred in order to distinguish into hypothetically life-long methylation patterns as a source of susceptibility to work-related stressor and dynamic molecular adaptions to recently experienced occupational adversities.

Another often raised controversy relates to the evaluation of epigenetic patterns in peripheral tissues, mainly leukocytes derived from blood or buccal cells from saliva samples, when the psychiatric main interest lies within the CNS pathophysiology of mental disorders. However, interindividual differences in brain methylome patterns have been proven to be reflected in peripheral samples (39), a positive cross-correlation between blood and saliva methylation has been confirmed (40), and both sampling types have been verified to deliver temporally stable results (41). Nonetheless, direct conclusions from peripheral to central molecular changes should be drawn with caution, and only independent replication and cross-tissue evidence will further support the rise of surrogate epigenetic biomarkers viable to contribute clinical benefits in therapeutic decision making.

Furthermore, accessible measures of daily life stress and work-related stress ultimately represent subjective values and are difficult to accurately quantify. The application of multiple validated retrospective self-report questionnaires assessing comparable stressor constructs, or of repeated recall bias-free real-time evaluations of behaviors and experiences in natural daily environments [ecological momentary assessment; (42)], appears to be the gold standard for future investigations. Also, epigenetic findings should be followed up by *in vivo* and *in vitro* evaluation of potential causal mechanisms relating transcriptomic expression changes to higher levels of biological functioning. Eventually, candidate risk gene will be combined into candidate risk haplotypes before hypothesis-driven work will start to complement increasingly accessible hypothesis-generating EWAS approaches (43). As this mini review has demonstrated, currently, there is a reliance on single-locus investigations and comparably small sample sizes (and therefore reduced statistical power), which has resulted in a research focus on mostly established candidate risk genes. Prospective multi-locus study designs will help to disentangle the epigenetic effects of occupational adversities and targeted protective measures in relation to work stress-related susceptibility (44).

### From Novel Pathophysiological Frameworks Toward Individualized Treatments

The present state of literature harmonizes with the notion of the “differential susceptibility hypothesis,” stressing that rather to hunt for »risk« genes, one should consider »plasticity« genes, which can render an individual more receptive to environmental stimuli in general, while their interaction can result in added allostatic load or buffer organismal defences (45). As exemplified above, the *5-HTT*LPR genotype-dependent *SLC6A4* promoter methylation in response to an acute (4) or chronic work-related stressor (6) might boost or diminish an individual’s subsequent reactions to similar or derivative forms of stress. Daily life stress, work-related stress, and SES therefore represent welcome additions to future epigenetic approaches to unravel the ever-elusive point where heredity and nature meet environment and nurture but should always be considered as plasticity variables and be evaluated alongside potential coping factors (46).

The accumulation and integration of multilevel evidence relating to individual epigenetic plasticity markers, for example, from SES-dependent modifications in *SLC6A4* promoter methylation to neuronal reactivity and finally to psychometric alterations (17), enhance the accessibility of predictive molecular signatures and finally deepen our insight into interlaced levels of systems biology (47). Biomarkers of disease course development or treatment response hold a particular value due to their inherent epigenetic dynamic. In light of potentially reversible SES-dependent maladaptive DNA methylation patterns, it should be noted that epigenetic markers have already been suggested as predictors and potential mechanistic correlates of pharmacological and psychological therapy responsiveness, with DNA methylation measurements reverting to values comparable to healthy controls over the time course of treatment (48–51). Multiple forms of preventive interventions against stressors like work exhaustion/strain and shift work and associated common mental disorders are currently recognized, including the enhancement of employee control and the promotion of physical activity (52). Future working generations might consequently possibly benefit from indicated preventive interventions or targeted treatment approaches depending on the quantifiable state of epigenetic biomarkers. Particularly, when considering potential long-term effects of transgenerational epigenetic inheritance and *in utero* exposure influenced by work-related stressors on mental health traits, which creates novel perspectives of social equality and responsibility alike (53).

## Conclusion

Notwithstanding the epidemiological and economic impact of workplace stressors, investigations into the relevant epigenetic mechanisms mediating susceptibility or resilience toward mental disorders have been limited. Gathered multilevel evidence specifically favors a role of *SLC6A4* promoter methylation patterns as correlates of acute daily stress, chronic work-related stress, and low SES upbringing, although hypothesis-generating studies supporting an involvement of the serotonergic system are still needed. Upcoming epigenetic research should aim to expand beyond an integration of occupational psychometric readouts as additional risk variables and progress to a dimensional understanding of psychopathology accompanied by prognostic molecular plasticity markers regulating individual work stress internalization and allowing for personalized treatment.

## Author Contributions

MG conducted the literature search and wrote the first manuscript draft. MS and KD contributed to the literature search and to the revision of the manuscript. All authors contributed to and have approved the final manuscript version.

## Funding

This work was partly supported by the Deutsche Forschungsgemeinschaft (DFG, German Research Foundation) – project number 44541416 – TRR 58 “Fear, Anxiety, Anxiety Disorders” (projects C02 and Z02 to KD), the German Ministry of Research and Education (BMBF, 01EE1402F, PROTECT-AD, project P5 to KD), and the EQUIP Medical Scientist Program of the Medical Faculty, University of Freiburg, Germany (to MAS). The funding sources had no further role in the study design, in the collection, analysis, and interpretation of data, in the writing of the report, and in the decision to submit the paper for publication.

## Conflict of Interest

KD is a member of the Janssen Pharmaceuticals, Inc. Steering Committee Neurosciences.

The remaining authors declare that the research was conducted in the absence of any commercial or financial relationships that could be construed as a potential conflict of interest.
